# Prevalences of Known and Presumed Inherited Eye Diseases in Pugs in Germany

**DOI:** 10.1111/vop.70235

**Published:** 2026-07-29

**Authors:** Carolin Lemle, Clara Koch, Andrea Meyer‐Lindenberg

**Affiliations:** ^1^ Clinic of Small Animal Surgery and Reproduction, Centre of Veterinary Clinical Medicine, Veterinary Faculty LMU Munich Germany

**Keywords:** brachycephalic ocular syndrome, DOK, entropion, hereditary cataract, keratoconjunctivitis sicca, pigmentary keratopathy

## Abstract

The aim of this retrospective study was to describe the prevalence and distribution of presumed inherited eye diseases in pugs in Germany and to evaluate potential risk factors for selected diseases. Therefore, ophthalmic findings from 294 pugs provided by the German panel of the European Eye Scheme programme were analyzed retrospectively. Associations between diseases and age and sex were assessed using multivariable linear probability models and correlation analyses and prevalences with 95% confidence intervals were reported. Entropion was the most frequent diagnosis (72.4%), followed by pigmentary keratopathy (36.7%), macroblepharon (24.8%), distichiasis (20.1%) and non‐congenital hereditary cataract (12.2%). Based on anatomic location, 81.3% of pugs had at least one adnexal disorder and 41.5% at least one corneal disorder. Increasing age was significantly associated with a higher probability of pigmentary keratopathy, keratoconjunctivitis sicca and hereditary cataract. No significant association with sex was identified. Pugs in this German population were commonly affected by adnexal and corneal diseases. Further ophthalmic screening and prospective studies are warranted to better characterize presumed hereditary eye diseases of this breed, clarify inheritance patterns, and improve ocular welfare in pugs.

**Abbreviations**
BOS: brachycephalic ocular syndrome
CAER: Companion Animal Eye Registry
CFR: craniofacial ratio
DOK: Dortmunder Kreis
ECVO: European College of Veterinary Ophthalmologists
ESE: European Eye Scheme Examiners
HC: hereditary cataract
HED: hereditary eye disease
ICAA: iridocorneal angle abnormality
KCS: keratoconjunctivitis sicca
PGNM: prolapsed gland of the nictitating membrane
PIED: presumed inherited eye disease
PK: pigmentary keratopathy
PPM: persistent pupillary membrane
PRA: progressive retinal atrophy
VDH: Verband für das Deutsche Hundewesen (German Kennel Club)

## 1 Introduction

Brachycephalic dogs such as the pug have become very popular in recent years [1, 2, 3, 4]. This popularity is mainly due to their appearance: their short snouts and large eyes visually correspond to an infant‐like facial appearance [5, 6]. Such head and facial conformation is associated with several serious health problems, including brachycephalic obstructive airway syndrome [7, 8, 9].

Also, ocular diseases related to brachycephaly have gained increasing attention among veterinarians within the last years [10, 11, 12, 13]. The term brachycephalic ocular syndrome (BOS) describes a combination of eye disorders that are directly or indirectly linked to brachycephaly [10, 12, 14]. The shallow orbits characteristic of brachycephalic breeds lead to pronounced globe protrusion, often accompanied by an enlarged palpebral fissure and incomplete eyelid closure. Further ocular and periocular disorders associated with the BOS complex include distichiasis, trichiasis, ectopic cilia, entropion, as well as reduced tear production and decreased corneal sensitivity [11, 15, 16]. These anatomical and physiological changes predispose these dogs to ocular surface disorders [10, 12, 14]. The risk of developing the latter increases as relative nasal length decreases [10]. Particularly disorders of the corneal surface, above all pigmentary keratopathy (PK), are very common in pugs [11, 17, 18]. However, it should be noted that the aforementioned conditions can occur individually or in combination, are not specific to BOS, and also can affect non‐brachycephalic breeds [11].

Most of the aforementioned eye diseases are considered as known or presumed to be inherited in various breeds [19, 20]. A condition is classified as a presumed inherited eye disease (PIED) when scientific evidence indicates a breed predisposition, the disease occurs more frequently among related dogs and demonstrates a characteristic clinical pattern with respect to age of onset, progression, or anatomical location. However, unlike hereditary eye diseases (HEDs), no DNA‐based diagnostic test is currently available [21, 22, 23].

For the pug, the following eye disorders are presumed to be inherited: entropion, macroblepharon, distichiasis, trichiasis, exposure keratopathy, keratoconjunctivitis sicca (KCS), pigmentary keratopathy (PK), epithelial corneal dystrophy, glaucoma, non‐congenital hereditary cataract (HC), persistent hyperplastic tunica vasculosa lentis/persistent hyperplastic primary vitreous (PHTVL/PHPV), retinal dysplasia (folds), persistent pupillary membranes (PPM), vitreous degeneration, and persistent hyaloid artery (PHA) [19, 23].

HEDs/PIEDs can lead to impaired vision or blindness, are often painful, and negatively affect the animals' well‐being [10, 20, 24, 25, 26]. Therefore, veterinarians have joined forces to establish Europe‐wide examination panels with the aim of controlling and preventing them. In Germany, the Dortmunder Kreis (DOK) serves as a national panel and collaborates closely with the European College of Veterinary Ophthalmologists (ECVO) [27, 28]. In addition to standardizing ophthalmic examinations for veterinarians and providing advisory services for breeding associations, it promotes data collection and encourages scientific research [29].

To the authors' knowledge, no scientific studies have been published to date on the occurrence of known and presumed hereditary eye diseases in Pugs in Germany. The aim of this retrospective study was to describe the prevalence and distribution of PIEDs in a population of Pugs in Germany. In addition, potential demographic risk factors including age and sex were evaluated for selected PIEDs.

## 2 Material and Methods

### 2.1 Study Design and Data Collection

All eye examination results for PIEDs in pugs were evaluated between June 2013 and March 2023. The data were provided by the DOK in Excel spreadsheet format. Solely Diplomates of the ECVO and DOK‐certified veterinarians, so‐called “European Eye Scheme Examiners” (ESE) [30], performed the ocular examinations in multiple veterinary clinics and practices across Germany.

Dogs examined for official breeding screening certification were referred to as breeding examination (BE) group, whereas dogs examined for reasons other than breeding purposes were assigned to the non‐breeding examination (NBE) group. The reasons for recording data on the second group were, on the one hand, data collection within the framework of the DOK and, on the other hand, for training purposes for ESE. The specific reasons for these pugs being brought to the veterinary practice or clinic—that is, whether they were brought in due to ocular issues or for other health reasons—were not documented. However, all eye examinations were carried out in a standardized manner and recorded on the ECVO/DOK Certificate, further referred to as the Eye Scheme [31, 32]. The individual ophthalmic examination results were categorized as unaffected, affected, or suspicious/undetermined [28, 31, 32]. The latter was the case when there were indications of PIED, but the result was inconclusive. Re‐examination was recommended after 12 months in adult dogs and after 3 months in puppies [31].

### 2.2 Data Processing and Transformation

Animal identification data were anonymised, and each animal was assigned a serial number. If a dog was examined several times during the period, the most recent examination results were used for the analysis. The animal‐related variables included in the statistical analysis were sex and age. Information on neuter status was not available. Considering the retrospective nature of the dataset, ophthalmic findings that were marked as suspicious/undetermined [31, 33] were coded as unaffected to enable a binary analysis. Prevalences of disorders marked as undetermined/suspicious are provided in Table S1. Information on whether a “severe” was ticked for any diagnosis in the HED certificate was not included in the collected data. No distinction was made between eyes affected unilaterally or bilaterally. If multiple disorders were diagnosed in the same eye, they were recorded as separate findings. Additionally, ocular disorders were categorized according to their anatomical localisation within the eye as follows: ocular adnexa, cornea, lens, uvea, vitreous body, fundus and globe. For the grouped disorders of ocular adnexa and cornea, each affected dog was counted only once per region, even if it had multiple diagnoses in the corresponding eye segment. These grouped prevalences therefore reflect the number of animals affected in the relevant eye segment, rather than the number of diagnoses. Dogs diagnosed with BOS were listed under the category *Other*, as it is a disease complex rather than a single diagnosis.

### 2.3 Statistical Analysis

The statistical analysis was performed using IBM SPSS Statistics (version 31). Descriptive statistics were calculated for the study population and eye disorders, including absolute and relative frequencies, as well as 95% confidence intervals. Unless otherwise stated, all percentages were calculated based on the total study population of 294 pugs. Mean and standard deviation were calculated for age. To account for the confounding effects of age, sex and purpose (breeding vs. non‐breeding), multivariable linear probability models were employed for each binary disease outcome (0/1), estimating changes in disease probability associated with each independent variable while holding the other covariates constant [34]. Categorical explanatory variables (sex and purpose) were included as binary dummy variables, with male and non‐breeding purpose serving as reference categories. Regression coefficients therefore represent differences in disease probability for sex and purpose, respectively, holding all other variables constant. The plausibility of predicted probabilities was checked to ensure values remained within the 0–1 range. For outcomes showing a significant relationship with age or sex, a multivariate analytical approach based on Pillai's Trace was applied to evaluate the global effect across multiple dependent variables simultaneously. All correlations were based on two‐sided hypothesis testing. A *p*‐value < 0.05 was set as the significance level for all statistical analyses.

## 3 Results

**FIGURE 1:**
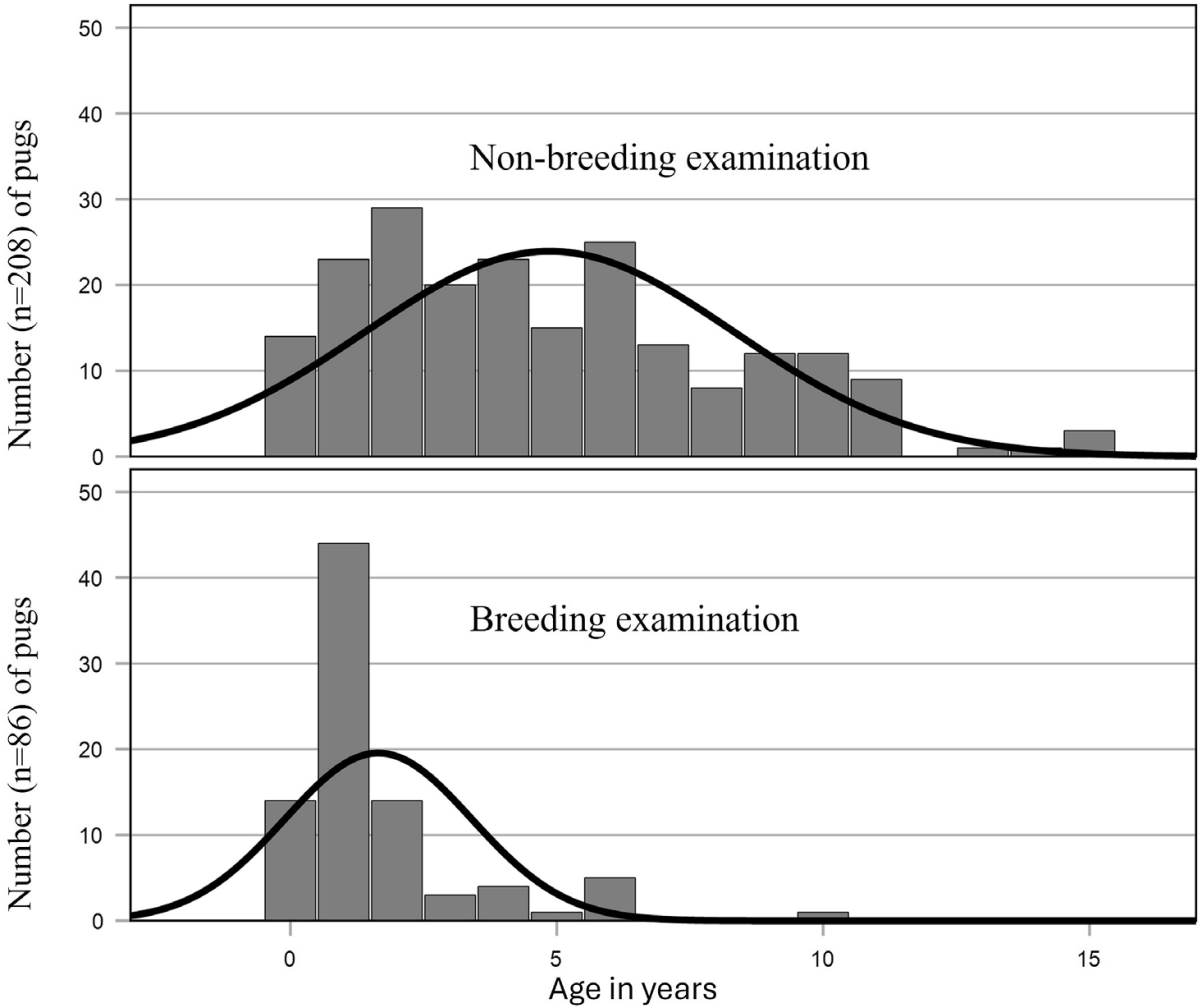
Age distribution of pugs (*n* = 294) according to the purpose of the ophthalmic examination (non‐breeding examination versus breeding examination). Bars represent the number of pugs per full year of age.

The data from 294 pugs were included in this study and are provided in Table S2. Of these, 154/294 (52.4%) were male and 140/294 (47.6%) were female. The mean age of the study population was 3.93 years (SD 3.40; range, 0–15 years). Overall, 208/294 pugs (70.8%) belonged to the NBE group, whereas 86/294 pugs (29.3%) were presented for official breeding examinations (BE). Dogs in the BE group were younger, with a mean age of 1.65 years (SD 1.75), compared with 4.87 years (SD 3.47) in the NBE group. For detailed age distribution see Figure 1.

**FIGURE 2:**
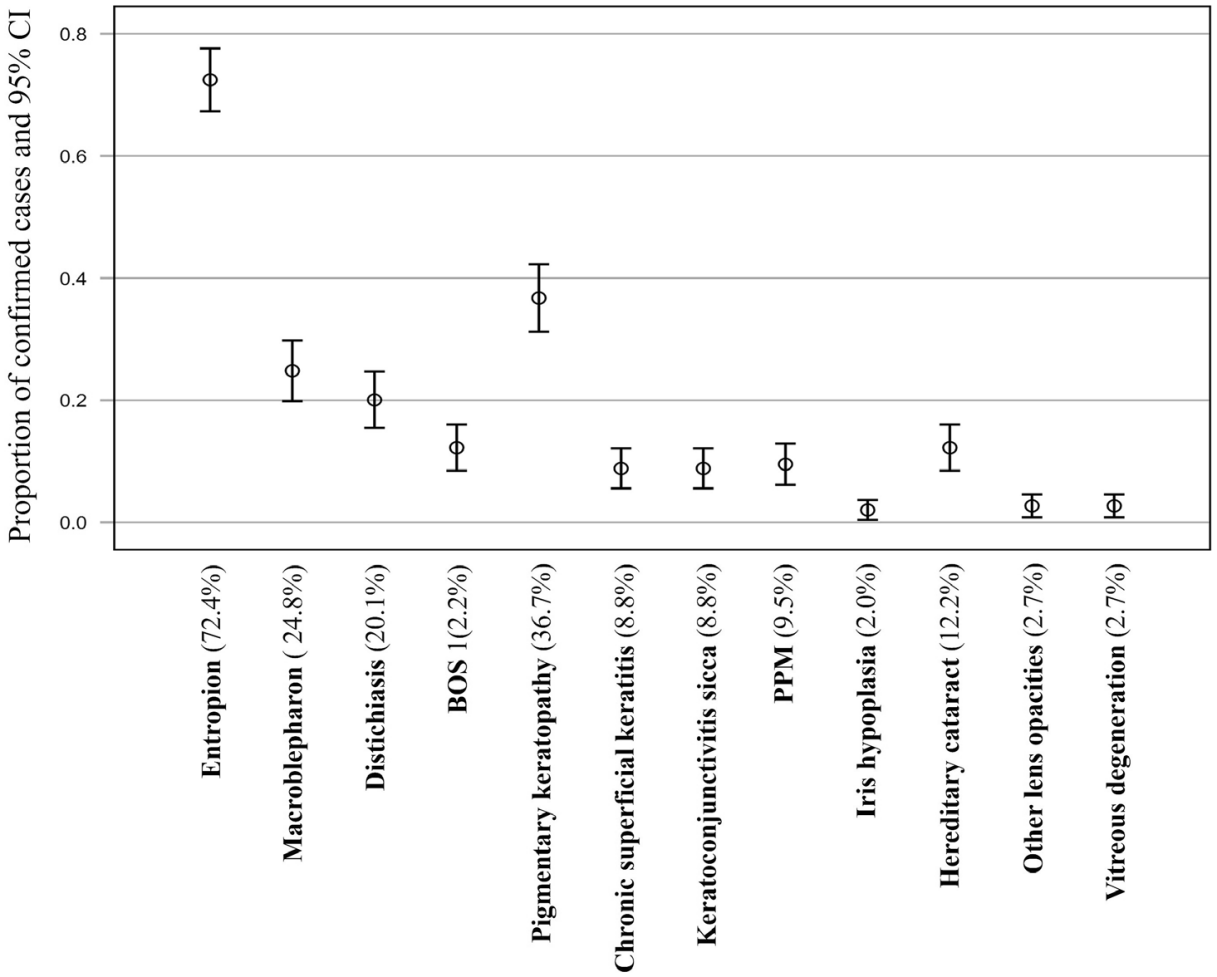
Percentage of examined pugs (*n* = 294) affected by each ophthalmic disease. Only diseases with more than five confirmed cases are shown. Error bars represent 95% confidence intervals.

Medial entropion associated with trichiasis was the most frequently diagnosed condition, confirmed in 213/294 pugs (72.4%, 95% CI 67.1%–77.3%), followed by PK, which was diagnosed in 108/294 dogs (36.7%, 95% CI 31.4%–42.4%). These two conditions were significantly more frequent than any other condition (Figure 2).

In all dogs diagnosed with entropion associated with trichiasis, the location of entropion was specified as medial. Additional to entropion, nasal fold trichiasis was described in 9 dogs, caruncular trichiasis in 6 dogs. Macroblepharon affected 73/294 dogs (24.8%, 95% CI 20.2%–30.0%), distichiasis affected 59/294 dogs (20.1%, 95% CI 15.8%–24.9%) and both, non‐congenital HC and BOS (12.2%, 95% CI 8.9%–16.4%) affected 36/294 pugs each. Of the 59/294 pugs with distichiasis, only one dog showed additional ectopic cilia. The localisation of HC was distributed as follows: cortical (*n* = 13), posterior polar (*n* = 8), nuclear (*n* = 3), cortical + nuclear (*n* = 4), cortical + posterior polar (*n* = 6) and cortical + posterior (*n* = 2). Further PIEDs diagnosed were PPM in 28/294 pugs (9.5%, 95% CI 6.6%–13.3%), KCS and chronic superficial keratitis (CSK) with each affecting 26/294 dogs (8.8%, 95% CI 6.0%–12.5%). Less common conditions affecting fewer than 10/294 animals were iris hypoplasia (2.0%), other lens opacities (2.7%), vitreous degeneration (2.7%), and globe abnormalities (1.0%). PIEDs with fewer than 5 pugs affected are listed in Table 1 under the respective anatomical location.

**TABLE 1:** Prevalence of PIEDs in pugs.

Location	Diagnostic name	*N* affected pugs	Percent and 95% CI
Ocular adnexa	Entropion	213	72.4 (67.1‐77.3)
	Macroblepharon	73	24.8 (20.2–30.0)
	Distichiasis	59	20.1 (15.8–24.9)
	PGNM	1	0.34
	Lacrimal punctum atresia	1	0.34
Cornea	Pigmentary keratopathy	108	36.7 (31.4–42.4)
	KCS	26	8.8 (6.0–12.5)
	CSK	26	8.8 (6.0–12.5)
Uvea	PPM	28	9.5 (6.6–13.3)
	Iris hypoplasia	6	2.0 (0.9–4.2)
	Choroidal coloboma	1	0.34
	Iris cyst	1	0.34
Lens	Hereditary cataract	36	12.2 (8.9–16.4)
	Lens luxation	1	0.34
	Other lens opacity	8	2.7 (1.3–5.1)
Vitreous body	Vitreous degeneration	8	2.7 (1.3–5.1)
	PHTVL	1	0.34
	Persistent hyaloid artery	3	1.0
Fundus	Retinal dysplasia (multifocal)	1	0.34
Globe	Macrophthalmos	2	0.68
	Microphthalmos	1	0.34
Other	BOS	36	12.2 (8.9–16.4)

*Note:* Prevalence of presumed inherited eye diseases (PIEDs) in pugs presented for ophthalmic examination (*n* = 294 dogs). Values represent the proportion of affected dogs within this population. The 95% confidence intervals (95% CIs) were calculated only for diagnoses with *n* ≥ 5 affected dogs.

Of the 294 pugs included in the study, 19/294 dogs were free of any PIED. When grouped by anatomical region, 239/294 dogs (81.3%, 95% CI 76.4%–85.4%) were affected by at least one adnexal disorder, and 122/294 (41.5%, 95% CI 36.0%–47.2%) had at least one disorder within the cornea. Diseases of the uvea (36/294; 12.2%, 95% CI 8.9%–16.4%), lens (44/294; 15.0%, 95% CI 11.2%–19.4%), vitreous body (11/294; 3.7%), globe (3/294; 1.0%), and fundus (1/294; 0.34%) were less frequently observed. However, as dogs could have abnormalities in more than one anatomical region, categories were not mutually exclusive.

Age was significantly associated with a higher probability of PK (*p* < 0.001), KCS (*p* < 0.001), and HC (*p* = 0.008), with the predicted probability increasing by 3.2, 2.6, and 1.7 percentage points per year of age, respectively. The multivariate analysis of variance demonstrated a significant overall effect of age (Pillai's Trace, *p* < 0.001).

Pugs of the NBE group had a significantly higher probability of being diagnosed with entropion/trichiasis (*p* = 0.005), macroblepharon (*p* = 0.009), or PK (*p* < 0.001). There was no significant association between sex and any of the analyzed PIEDs.

A significant association was identified between PK and the presence of adnexal and corneal diseases. The distribution of these disorders differed between pugs with and without PK (Cramér's V = 0.385, *p* < 0.001). Among dogs affected by PK, concurrent adnexal and corneal disorders were present in 79%. No significant correlations were detected between pigmentary keratopathy and iris hypoplasia or persistent pupillary membrane.

Macroblepharon was more prevalent in dogs with entropion than in those without (29.6% vs. 12.3%). Similarly, entropion occurred in 86.3% of dogs with macroblepharon compared to 67.9% without. A weak but significant positive correlation was found between both conditions (*r* = 0.178, *p* = 0.002).

## 4 Discussion

This is the first retrospective cross‐sectional study of presumed hereditary eye diseases in pugs in Germany over a 10‐year period. The most frequently diagnosed PIEDs were entropion, PK, macroblepharon, distichiasis, HC, and BOS, the latter two occurring with equal prevalence. Medial entropion of the lower eyelid typically occurs in brachycephalic breeds and is usually associated with an abnormally short medial canthal ligament [15, 35, 36].

In our study, medial entropion associated with trichiasis was the most common PIED, with a prevalence of 72% in the study population. In a study by Costa et al., entropion was also the most common eyelid malformation in dogs diagnosed with BOS [11]. In an Austrian study by Krecny et al., all pugs of their study population were affected by nasal entropion and macroblepharon [37]. Similarly, we found a weak but significant correlation between entropion and macroblepharon, with macroblepharon being more prevalent in dogs with entropion and vice versa. This may indicate a tendency toward co‐occurrence, related to the breed's conformational peculiarity.

Macroblepharon was present in nearly 25% of the study population. This relatively high prevalence is not surprising, as brachycephalic dog breeds often exhibit excessively long and rounded eyelids, frequently in combination with exophthalmos due to shallow orbits [31, 35, 38]. In the American College of Veterinary Ophthalmologists' (ACVO) breed report, known as the ‘Blue Book’, none of the nearly 1000 pugs examined between 2019 and 2023 were diagnosed with macroblepharon [23]. However, the higher prevalence of macroblepharon observed in the present study may partly reflect differences between certification schemes. Macroblepharon is a specific diagnostic category within the European Eye Scheme [31], whereas it is not listed as a separate diagnosis on the American Companion Animal Eye Registry (CAER) certification [39], potentially limiting direct comparisons between studies. Additionally, there might also be variation in breeding lines, referral bias, and possible under‐recognition of mild forms that may be interpreted as normal brachycephalic conformation.

Distichiasis was found in 20% of the pugs. Other brachycephalic breeds also appear to be predisposed to this condition [11, 40, 41]. Although distichiasis and ectopic cilia are anatomically and developmentally related disorders [42], only one pug with distichiasis in the present study had concurrent ectopic cilia.

This contradicts the assumption that dogs with distichiasis are predisposed to developing ectopic cilia [42, 43] and may reflect breed‐specific differences. However, due to the retrospective study design, previously removed ectopic cilia may not have been recorded, potentially leading to an underestimation of their occurrence.

Most dogs (81%) showed at least one disorder of the ocular adnexa. Diseases of the ocular surface were also common, with 122 pugs affected by at least one condition in this area.

Of these, PK was the second most frequent PIED, accounting for 108 dogs. This high prevalence is consistent with previous studies in other countries, where the pug has been found highly affected by this entity [17, 37, 44, 45, 46, 47]. PK can develop due to chronic irritation and is often associated with chronic keratitis [44, 48]. Possible multiple underlying causes or contributing factors such as distichiasis, nasal fold trichiasis, medial lower entropion, macroblepharon, and tear film abnormalities such as KCS can lead to this condition [37, 45, 47, 48, 49]. This is in line with the findings of the present study, in which most pugs with PK had concurrent adnexal and corneal disorders. Regarding age, findings are inconsistent. While this study identified a significant association between age and PK (3.2% per year) and thus corroborates previous findings [17, 18, 45, 47], others found no such relationship [37, 44]. There was no significant association between PPM or iris hypoplasia and PK in this study. Although a significant correlation between these conditions and PK has not been reported to date, some authors have reported a high proportion of pugs with PK to have concurrent PPMs or iris hypoplasia [44, 47]. These discrepancies suggest that concurrent ocular abnormalities may contribute to the development or exacerbation of PK; however, the exact underlying mechanisms remain unclear.

A possible genetic basis of PK is suspected by several authors [37, 44, 47] as well as potential associations between uveal disorders and PK that need to be further investigated.

KCS was identified in 8.8% of pugs. The pug is one of the predisposed breeds for KCS [18, 50, 51]. Generally, brachycephalic breeds have been reported to have lower aqueous tear film production and reduced corneal sensitivity than mesocephalic breeds, which may contribute to their increased susceptibility to this disorder [16, 50]. Depending on the underlying cause, KCS can occur at any age [52]. Sansom and Barnett reported an average age of 5 years at initial presentation, regardless of breed [53]. Aguirre et al. described a biphasic age distribution with peaks at 1 and 4 years [54]. Overall, the likelihood of developing KCS increases with age [17, 50], which agrees with the results of the present study. No sex predisposition was found although previous findings of possible sex predispositions in KCS have been inconsistent [50, 54, 55, 56]. Similar to KCS, CSK was diagnosed in 8.8% of dogs. CSK, also called Pannus or Uberreiter's syndrome, is commonly seen in German Shepherds and Greyhounds [48]. The pug is not mentioned as a predisposed breed in the literature yet. The relatively high prevalence of CSK in this study may be due to the breed's susceptibility to ocular surface diseases. However, it cannot be excluded that some cases diagnosed as CSK may have represented secondary exposure keratitis resulting from excessive globe exposure due to shallow orbits, lagophthalmos, and/or tear film disorders, rather than primary immune‐mediated CSK.

Overall, 81% of examined pugs were affected by at least one disorder of the ocular adnexa, and 42% had at least one disorder of the corneal surface. Many of these conditions are probably related to the breed's ocular and facial conformation [10, 12, 20].

Despite the high prevalence of adnexal and corneal disorders in pugs, only 12% of the pugs were classified as having BOS. Because BOS represents a symptom complex, it should be interpreted as an overlapping syndrome classification. The severity of the individual BOS‐associated abnormalities was not assessed in this study. Therefore, mild combinations of these features may not have been assigned an additional diagnosis of BOS.

Non‐congenital HCs are the most common causes of vision impairment and vision loss in purebred dogs and have been identified in 180 breeds to date [25, 26]. HCs represent the most prevalent ophthalmic disorder reported to date in dogs in North America. In the present study, 12% of pugs were diagnosed with HC. By comparison, Krecny et al. reported a prevalence of 8% in examined pugs in Austria, whereas Guandalini et al. found even higher prevalences, with HC affecting 25% of Maltese and 19% of Bolognese dogs in their study population [57]. Palmer et al. likewise reported a high frequency of cataracts in pugs, as well as in Shih Tzus and Boston Terriers [17]. In contrast, Gelatt et al. documented a lower prevalence in a North American study, with primary cataracts affecting 2.5% of pugs. The varying frequencies of HC in pugs might be due to variations in diagnostic criteria but also by geographic differences in breeding lines. It is presumed that a mutation in the *HSF4* gene, known to cause early HC in Staffordshire Bull Terriers and Boston Terriers, may also play a role in French Bulldogs [58]. However, no reports are available for pugs and the inheritance mode of HC is still unknown [19]. Increasing age was associated with a higher probability of HC in this study population. Senile cataracts are typically observed in dogs older than 9 years and should be distinguished from HCs, although this distinction is not always straightforward [59, 60].

The associations between age and the diagnosis of HC, but also of PK and KCS, are particularly relevant when interpreting the differences between the BE and NBE groups. Dogs presented for breeding examinations were markedly younger than dogs in the NBE group; therefore, later‐onset PIEDs may have been underdiagnosed in the BE group at the time of examination. This is of concern for breeding decisions, as dogs may be classified as unaffected during a young‐age screening examination but may develop clinically relevant ocular disease later in life, potentially after they have already been used for breeding. Therefore, a single ophthalmic examination before breeding may be insufficient to detect conditions such as HC, PK, and KCS, which can occur at any age [17, 18, 26, 45, 47, 50, 59]. Repeated ophthalmic screening throughout the breeding period and later in life may help identify affected dogs more reliably and support breeding strategies aimed at reducing the prevalence of ocular disease in pugs. The effectiveness of this measure was demonstrated in a study by Koll et al., which reported a decreasing prevalence of HC in German Dachshunds [61].

Data on lens and fundus abnormalities in pugs are limited. In the present study, only one pug was affected by a retinal disease, namely multifocal retinal dysplasia. In contrast, a North American study on Sudden acquired retinal degeneration cases reported that pugs accounted for 8% of all confirmed cases [62]. To obtain more data on PIEDs in this breed, especially regarding the lens and the posterior eye segment, mandatory eye examinations should be introduced for this breed. Recently, a breed club affiliated with the German Kennel Club introduced mandatory breeding eye examinations for pugs, representing an important step toward improving the ocular health of this breed [63].

There are limitations of this study which should be considered when interpreting the results. The retrospective nature of data collection introduces a risk of multiple biases. Only pugs examined by ESE or Diplomates were included. Consequently, the examined population may over‐ or underrepresent certain conditions. Although potential confounding by age and examination purpose (NBE vs. BE group) was included as covariates in the multivariable models, residual selection bias cannot be excluded. Furthermore, suspicious/undetermined diagnoses were retrospectively coded as unaffected and may have led to underestimation of disease prevalences. Regarding the 10‐year study period, this may have introduced bias through changes in breeding selection, conformational trends or increased owner awareness. In addition, the evolvement of the Scheme may have affected the interpretation and recording of ocular abnormalities during the study period. Because retrospective reclassification according to a single manual version was not possible, this may have influenced the reported prevalence of some findings. And finally, the overall population consisted mainly of young and middle‐aged dogs, thus age‐related ocular disorders could also be underestimated. Despite these limitations, this study benefits from its standardized examination and documentation according to the Scheme, carried out by specialized and trained veterinarians and Diplomates. This likely ensured high internal validity and minimized inter‐observer variability.

## 5 Conclusion

This is the first comprehensive report on presumed inherited eye diseases in pugs in Germany.

The high prevalences of adnexal and corneal disorders likely reflect the direct and indirect consequences of this breed's exaggerated facial conformation. The implementation of mandatory breeding eye examinations could help raise awareness of the negative ocular effects associated with brachycephalic morphology, as well as of other PIEDs in this breed. Further prospective studies are warranted to better characterize PIEDs in pugs, clarify their inheritance patterns, and evaluate whether breeding away from exaggerated facial conformation improves ocular health and welfare.

## Author Contributions

**Andrea Meyer‐Lindenberg:** conceptualization, writing – review and editing, supervision, methodology, investigation, project administration, resources. **Clara Koch:** writing – review and editing, investigation, supervision. **Carolin Lemle:** conceptualization, methodology, data curation, investigation, validation, writing – original draft, writing – review and editing.

## Funding

The authors have nothing to report.

## Disclosure

The authors have not used AI to generate any part of the manuscript.

## Ethics Statement

This study was approved by the Head of the Clinic of Small Animal Surgery and Reproduction LMU Munich and by the Ethics Committee of the Faculty of Veterinary Medicine, LMU Munich, under file reference no. 437‐07‐02‐25. Clinical ophthalmic examinations were performed as part of clinical routine with the consent of the dog owners, including further data analysis of the results. Written owner consent was obtained by signing the DOK standard examination form and, in the case of breeding animals, the Kennel Club individual examination form. The use of the pooled DOK data for statistical analysis in this study is authorized by the DOK, through its elected board members.

## Conflicts of Interest

The authors declare no conflicts of interest.

## Supporting information


**Table S1:** Suspicious/undetermined PIEDs in all examined pugs (*n* = 294).

**Table S2:** Anonymized dataset of all examined pugs (*n* = 294).

## Data Availability

The data that supports the findings of this study are available in the Supporting Information of this article.

## References

[1] Z. Bognár and E. Kubinyi , “The Brachycephalic Paradox: The Relationship Between Attitudes, Demography, Personality, Health Awareness, and Dog‐Human Eye Contact,” Applied Animal Behaviour Science 264 (2023): 105948.

[2] K. T. Teng , P. D. McGreevy , J. A. L. M. L. Toribio , and N. K. Dhand , “Trends in Popularity of Some Morphological Traits of Purebred Dogs in Australia,” Canine Genetics and Epidemiology 3, no. 1 (2016): 2. 27051522 10.1186/s40575-016-0032-2 PMC4820977

[3] A. K. Club , “The Most Popular Dog Breeds of 2023,” (2022), https://www.akc.org/expert‐advice/dog‐breeds/most‐popular‐dog‐breeds‐2022/.

[4] A. K. Club , “The Most Popular Dog Breeds of 2024,” (2025), https://www.akc.org/expert‐advice/news/most‐popular‐dog‐breeds‐2024/.

[5] R. M. A. Packer , D. G. O'Neill , F. Fletcher , and M. J. Farnworth , “Come for the Looks, Stay for the Personality? A Mixed Methods Investigation of Reacquisition and Owner Recommendation of Bulldogs, French Bulldogs and Pugs,” PLoS One 15, no. 8 (2020): e0237276. 32845902 10.1371/journal.pone.0237276 PMC7449392

[6] E. S. Paul , R. M. A. Packer , P. D. McGreevy , E. Coombe , E. Mendl , and V. Neville , “That Brachycephalic Look: Infant‐Like Facial Appearance in Short‐Muzzled Dog Breeds,” Animal Welfare 32 (2023): e5. 38487431 10.1017/awf.2022.6 PMC10936394

[7] R. M. Packer , A. Hendricks , M. S. Tivers , and C. C. Burn , “Impact of Facial Conformation on Canine Health: Brachycephalic Obstructive Airway Syndrome,” PLoS One 10, no. 10 (2015): e0137496. 26509577 10.1371/journal.pone.0137496 PMC4624979

[8] D. G. O'Neill , L. Baral , D. B. Church , D. C. Brodbelt , and R. M. A. Packer , “Demography and Disorders of the French Bulldog Population Under Primary Veterinary Care in the UK in 2013,” Canine Genetics and Epidemiology 5 (2018): 13. 10.1186/s40575-018-0057-9 PMC5932866 29750111

[9] D. G. O'Neill , C. Pegram , P. Crocker , D. C. Brodbelt , D. B. Church , and R. M. A. Packer , “Unravelling the Health Status of Brachycephalic Dogs in the UK Using Multivariable Analysis,” Scientific Reports 10, no. 1 (2020): 17251. 33057051 10.1038/s41598-020-73088-y PMC7560694

[10] R. M. Packer , A. Hendricks , and C. C. Burn , “Impact of Facial Conformation on Canine Health: Corneal Ulceration,” PLoS One 10, no. 5 (2015): e0123827. 25969983 10.1371/journal.pone.0123827 PMC4430292

[11] J. Costa , A. Steinmetz , and E. Delgado , “Clinical Signs of Brachycephalic Ocular Syndrome in 93 Dogs,” Irish Veterinary Journal 74, no. 1 (2021): 3. 33494828 10.1186/s13620-021-00183-5 PMC7836154

[12] L. Sebbag and R. F. Sanchez , “The Pandemic of Ocular Surface Disease in Brachycephalic Dogs: The Brachycephalic Ocular Syndrome,” Veterinary Ophthalmology 26, no. Suppl 1 (2023): 31–46. 10.1111/vop.13054 36585820

[13] H. Iwashita , S. Wakaiki , Y. Kazama , and A. Saito , “Breed Prevalence of Canine Ulcerative Keratitis According to Depth of Corneal Involvement,” Veterinary Ophthalmology 23, no. 5 (2020): 849–855. 32716142 10.1111/vop.12808

[14] D. Nutbrown‐Hughes , “Brachycephalic Ocular Syndrome in Dogs,” Companion Animal 26, no. 5 (2021): 1–9.

[15] S. Bettenay , R. S. Mueller , and D. J. Maggs , “Diseases of the Eyelids,” in Slatter's Fundamentals of Veterinary Ophthalmology, 6th ed. P. E. M. D. J. Maggs and R. Ofri (Elsevier, 2018), 127–134.

[16] H. Bolzanni , A. P. Oriá , A. C. S. Raposo , and L. Sebbag , “Aqueous Tear Assessment in Dogs: Impact of Cephalic Conformation, Inter‐Test Correlations, and Test‐Retest Repeatability,” Veterinary Ophthalmology 23, no. 3 (2020): 534–543. 32162773 10.1111/vop.12751

[17] S. V. Palmer , F. Espinheira Gomes , and J. A. A. McArt , “Ophthalmic Disorders in a Referral Population of Seven Breeds of Brachycephalic Dogs: 970 Cases (2008‐2017),” Journal of the American Veterinary Medical Association 259, no. 11 (2021): 1318–1324. 34727059 10.2460/javma.20.07.0388

[18] W. I. Lau and R. M. Taylor , “The Prevalence of Corneal Disorders in Pugs Attending Primary Care Veterinary Practices in Australia,” Animals (Basel) 15, no. 4 (2025): 531. 40003013 10.3390/ani15040531 PMC11851709

[19] ECVO HED Committee , “ECVO Manual Chapter 9—Canine Breeds,” (2021), https://www.ecvo.eu/hereditary‐eye‐diseases/ecvo‐manual/chapter‐9‐breeds‐dogs.html.

[20] K. C. Barnett , “Inherited Eye Disease in the Dog and Cat,” Journal of Small Animal Practice 29, no. 7 (1988): 462–475.

[21] ECVO HED Committee , “ECVO Manual Chapter 5—Definitions,” (2023), https://www.ecvo.eu/media/chapter_5_definitions_2023.pdf.

[22] ECVO HED Committee , “ECVO Manual Chapter 8—The Veterinary Ophthalmologists' Advice,” (2024).

[23] ACVO Genetics Committee , The Blue Book. Ocular Disorders Presumed To Be Inherited in Purebred Dogs (ACVO Genetics Committee, 2023), 458–462.

[24] D. G. O'Neill , M. M. Lee , D. C. Brodbelt , D. B. Church , and R. F. Sanchez , “Corneal Ulcerative Disease in Dogs Under Primary Veterinary Care in England: Epidemiology and Clinical Management,” Canine Genetics and Epidemiology 4 (2017): 5. 28630713 10.1186/s40575-017-0045-5 PMC5471714

[25] M. Leiva and T. Pena , “Diseases of the Lens and Cataract Formation,” in Veterinary Ophthalmology, ed. K. N. Gelatt (John Wiley & Sons, Inc., 2021), 1317–1370.

[26] K. N. Gelatt and E. O. Mackay , “Prevalence of Primary Breed‐Related Cataracts in the Dog in North America,” Veterinary Ophthalmology 8, no. 2 (2005): 101–111. 15762923 10.1111/j.1463-5224.2005.00352.x

[27] ECVO HED Committee , “ECVO Manual Chapter 3—The ECVO Hereditary Eye Disease Scheme,” (2024), https://www.ecvo.eu/media/chapter_3_eye_scheme_2024.pdf.

[28] DOK Dortmunder Kreis , Gesellschaft für Diagnostik Genetisch Bedingter Augenerkrankungen bei Tieren e.V (Dortmunder Kreis, 2024).

[29] DOK Dortmunder Kreis , “Wer Sind Wir,” (2019), https://www.dok‐vet.de/Pub/Wer%20sind%20wir.aspx?t=1.

[30] ECVO HED Committee , “ECVO Manual Chapter 2—Introduction,” (2013), https://www.ecvo.eu/media/2‐introduction.pdf.

[31] ECVO HED Committee , “ECVO Manual Chapter 6—Guidelines,” (2024), https://www.ecvo.eu/media/chapter_6_guidelines_2024.pdf.

[32] ECVO HED Committee , “ECVO Manual Chapter 4—The ECVO Certificate,” (2024), https://www.ecvo.eu/media/ecvo_hed_certificate_‐_for_practice_purposes_only.pdf.

[33] DOK Dortmunder Kreis , “Gesellschaft für Diagnostik Genetisch Bedingter Augenerkrankungen bei Tieren e.V.,” (2019), https://www.dok‐vet.de/Pub/Untersuchung/Befundbogen.aspx.

[34] J. M. Wooldridge , Econometric Analysis of Cross Section and Panel Data, 2nd ed. (MIT Press, 2010).

[35] S. Sahr , I. Clasen , and A. Steinmetz , “Makroblepharon—Eine Häufige Lidanomalie beim Hund,” Kleintier Konkret 16, no. 4 (2013): 28–35.

[36] N. Y. Yi , S. A. Park , M. B. Jeong , et al., “Medial Canthoplasty for Epiphora in Dogs: A Retrospective Study of 23 Cases,” Journal of the American Animal Hospital Association 42, no. 6 (2006): 435–439. 17088390 10.5326/0420435

[37] M. Krecny , A. Tichy , J. Rushton , and B. Nell , “A Retrospective Survey of Ocular Abnormalities in Pugs: 130 Cases,” Journal of Small Animal Practice 56, no. 2 (2015): 96–102. 25370448 10.1111/jsap.12291

[38] N. Rujirekasuwan , P. Sattasathuchana , W. Theerapan , and N. Thengchaisri , “Comparative Analysis of Ocular Biometry, Ocular Protrusion, and Palpebral Fissure Dimensions in Brachycephalic and Nonbrachycephalic Dog Breeds,” Veterinary Radiology & Ultrasound 65, no. 4 (2024): 437–446. 38682866 10.1111/vru.13351

[39] K. A. Diehl , S. K. Asif , and F. Mowat , “Ophthalmic Disease and Screening in Breeding Dogs,” Veterinary Clinics of North America: Small Animal Practice 53, no. 5 (2023): 965–983. 37246013 10.1016/j.cvsm.2023.04.003 PMC10527272

[40] C. Jondeau , M. Gounon , A. Bourguet , and S. Chahory , “Epidemiology and Clinical Significance of Canine Distichiasis: A Retrospective Study of 291 Cases,” Veterinary Ophthalmology 26, no. 4 (2023): 339–346. 37028946 10.1111/vop.13091

[41] K. K. L. Bellamy , F. Lingaas , and P. Madsen , “Heritability of Distichiasis in Havanese Dogs in Norway,” Canine Medicine and Genetics 8, no. 1 (2021): 11. 34784963 10.1186/s40575-021-00110-5 PMC8594152

[42] F. C. Stades and A. Woerdt , “Diseases and Surgery of the Canine Eyelid,” in Veterinary Ophthalmology, ed. K. N. Gelatt (John Wiley & Sons Inc, 2021), 923–987.

[43] T. Dulaurent , A. M. Dulaurent , I. Mathieson , et al., “Ectopic Cilia in 112 Dogs: A Multicenter Retrospective Study,” Veterinary Ophthalmology 25, no. 2 (2022): 186–190. 34821455 10.1111/vop.12947

[44] A. L. Labelle , C. B. Dresser , R. E. Hamor , M. C. Allender , and J. L. Disney , “Characteristics of, Prevalence of, and Risk Factors for Corneal Pigmentation (Pigmentary Keratopathy) in Pugs,” Journal of the American Veterinary Medical Association 243, no. 5 (2013): 667–674. 23971846 10.2460/javma.243.5.667

[45] S. Maini , R. Everson , C. Dawson , Y. M. Chang , C. Hartley , and R. F. Sanchez , “Pigmentary Keratitis in Pugs in the United Kingdom: Prevalence and Associated Features,” BMC Veterinary Research 15, no. 1 (2019): 384. 31666065 10.1186/s12917-019-2127-y PMC6822449

[46] L. V. Vallone , A. M. Enders , H. O. Mohammed , and E. C. Ledbetter , “In Vivo Confocal Microscopy of Brachycephalic Dogs With and Without Superficial Corneal Pigment,” Veterinary Ophthalmology 20, no. 4 (2017): 294–303. 27468727 10.1111/vop.12416

[47] D. Sarmiento Quintana , I. Morales Fariña , J. González Pérez , J. R. Jaber , and J. A. Corbera , “Ocular Surface Characteristics in Pugs With Pigmentary Keratitis in the Canary Islands, Spain,” Animals (Basel) 14, no. 4 (2024): 580. 38396548 10.3390/ani14040580 PMC10885891

[48] D. R. Whitley and R. E. Hamor , “Diseases and Surgery of the Canine Cornea and Sclera,” in Veterinary Ophthalmology, ed. K. N. Gelatt (John Wiley & Sons, 2021), 1082–1172.

[49] C. Pascual , “Pigmentary Keratitis in Dogs,” Indian Journal of Veterinary Research 24 (2015): 31–33.

[50] D. G. O'Neill , D. C. Brodbelt , A. Keddy , D. B. Church , and R. F. Sanchez , “Keratoconjunctivitis Sicca in Dogs Under Primary Veterinary Care in the UK: An Epidemiological Study,” Journal of Small Animal Practice 62, no. 8 (2021): 636–645. 34134171 10.1111/jsap.13382

[51] S. Chaithra , A. Gopinathan , K. Singh , et al., “Epidemiological Insights Into Keratoconjunctivitis Sicca (KCS) in Canine Populations: A Study in Brachycephalic Breeds,” Journal of Experimental Zoology, India 27 (2024): 1921–1922.

[52] H. D. Westermeyer , D. A. Ward , and K. Abrams , “Breed predisposition to congenital alacrima in dogs,” Veterinary Ophthalmology 12, no. 1 (2009): 1–5. 10.1111/j.1463-5224.2009.00665.x 19152591

[53] J. Sansom and K. C. Barnett , “Keratoconjunctivitis Sicca in the Dog: A Review of Two Hundred Cases,” Journal of Small Animal Practice 26, no. 3 (1985): 121–131.

[54] G. D. Aguirre , L. F. Rubin , and C. E. Harvey , “Keratoconjunctivitis Sicca in Dogs,” Journal of the American Veterinary Medical Association 158, no. 9 (1971): 1566–1579. 5104964

[55] R. L. Kaswan , C. L. Martin , and D. L. Dawe , “Keratoconjunctivitis Sicca: Immunological Evaluation of 62 Canine Cases,” American Journal of Veterinary Research 46, no. 2 (1985): 376–383. 3888007

[56] R. F. Sanchez , G. Innocent , J. Mould , and F. M. Billson , “Canine Keratoconjunctivitis Sicca: Disease Trends in a Review of 229 Cases,” Journal of Small Animal Practice 48, no. 4 (2007): 211–217. 17381766 10.1111/j.1748-5827.2006.00185.x

[57] A. Guandalini , N. di Girolamo , R. Corvi , D. Santillo , V. Andreani , and B. Pinzo , “Epidemiology of Ocular Disorders Presumed To Be Inherited in Three Small Italian Dog Breeds in Italy,” Veterinary Ophthalmology 21, no. 5 (2018): 524–529. 29284193 10.1111/vop.12542

[58] C. S. Mellersh , B. McLaughlin , S. Ahonen , L. Pettitt , H. Lohi , and K. C. Barnett , “Mutation in HSF4 Is Associated With Hereditary Cataract in the Australian Shepherd,” Veterinary Ophthalmology 12, no. 6 (2009): 372–378. 19883468 10.1111/j.1463-5224.2009.00735.x

[59] D. L. Williams , M. F. Heath , and C. Wallis , “Prevalence of Canine Cataract: Preliminary Results of a Cross‐Sectional Study,” Veterinary Ophthalmology 7, no. 1 (2004): 29–35. 14738504 10.1111/j.1463-5224.2004.00317.x

[60] S. R. Urfer , K. Greer , and N. S. Wolf , “Age‐Related Cataract in Dogs: A Biomarker for Life Span and Its Relation to Body Size,” Age (Dordrecht, Netherlands) 33, no. 3 (2011): 451–460. 20607428 10.1007/s11357-010-9158-4 PMC3168595

[61] S. Koll , S. Reese , I. Medugorac , C. U. Rosenhagen , R. F. Sanchez , and R. Köstlin , “The Effect of Repeated Eye Examinations and Breeding Advice on the Prevalence and Incidence of Cataracts and Progressive Retinal Atrophy in German Dachshunds Over a 13‐Year Period,” Veterinary Ophthalmology 20, no. 2 (2017): 114–122. 27073021 10.1111/vop.12378

[62] A. R. Heller , A. van der Woerdt , J. E. Gaarder , et al., “Sudden Acquired Retinal Degeneration in Dogs: Breed Distribution of 495 Canines,” Veterinary Ophthalmology 20, no. 2 (2017): 103–106. 26938661 10.1111/vop.12370

[63] Club für den Mops e.V ., “Aktuelle Zuchtinformationen,” (2025), https://www.cfd‐mops.de/aktuelles.html.

