# Supplementary material for: Prevalences of Known and Presumed Inherited Eye Diseases in Pugs in Germany
Source: Vet Ophthalmol. 2026 Jul 29;29(5):e70235. doi: 10.1111/vop.70235 (PMC13420794; doi:10.1111/vop.70235)
Supplement: Supplementary file 1 — Table S1: Suspicious/undetermined PIEDs in all examined pugs (n = 294). [file VOP-29-0-s002.docx]

S1. Suspicious and undetermined PIEDs in examined pugs (n = 294)

| **Anatomical region** | **PIED** | **Classification** | **Number of pugs** |
| --- | --- | --- | --- |
|  |  |  |  |
| *Ocular adnexa* | Entropion | undetermined | 1 |
|  | Macroblepharon |  |  |
|  | Distichiasis |  |  |
|  | PGNM |  |  |
|  | Lacrimal punctum atresia |  |  |
|  |  |  |  |
| *Cornea* | Cornea dystrophy | undetermined | 2 |
|  | KCS |  |  |
|  | CSK |  |  |
| *Uvea* | PPM | suspicious | 2 |
|  |  |  |  |
| *Lens* | Hereditary cataract | undetermined | 1 |
|  | Lens luxation | undetermined | 2 |
|  | congenital cataract | suspicious | 2 |
|  |  |  |  |
| *Vitreous body* | PHTVL | suspicious | 3 |
|  |  |  |  |
| *Fundus* | Retinal dysplasia | suspicious | 1 |
|  | PRA | undetermined | 1 |
|  | CEA | suspicious | 1 |
|  | Hypoplasie/Micro papilla | suspicious | 1 |
|  |  |  |  |
| *Others* | Multiple ocular anomalies | suspicious | 1 |
|  |  |  |  |
